# Collaborative Micro-Practices of Expert Healthcare Dyads: Implications for Medical Education

**DOI:** 10.5334/pme.1932

**Published:** 2026-01-20

**Authors:** Katie Walker, Maryam Asoodar, Michael Meguerdichian, Michaela Kolbe, Jenny Rudolph, Pim Teunissen

**Affiliations:** 1 School of Health Professions Education, Maastricht University, Australia; 2 Faculty, School of Health Professions Education, Maastricht University, Maastricht, Netherlands; 3 Simulation, NYC Health+Hospitals, New York City, New York, USA; 4 Simulation Center, University Hospital Zurich, Switzerland; 5 Center for Medical Simulation, Boston, USA; 6 School of Health Professions Education, Maastricht University, Maastricht, Netherlands

## Abstract

**Introduction:**

Despite widespread investment in teamwork training, coordination failures persist in acute healthcare environments. Traditional team-based education tends to focus on large teams or isolated technical skills, often overlooking the smallest and arguably most critical unit of collaboration: the healthcare dyad. This study explored how expert healthcare dyads; two individuals working closely in high-stakes clinical settings developed and sustained collaborative expertise. Furthermore, we considered how their practices might have an impact on health professions education.

**Methods:**

We conducted a limited realist-perspective study comprising 10 semi-structured dyadic interviews (20 participants) of expert healthcare dyads in acute care settings. Participants were purposively sampled. Using template analysis, we began with a preliminary coding template based on relational coordination and distributed cognition, then iteratively revised it. We undertook deductive indexing using the template, followed by open coding of uncaptured data. Codes were charted, and our analytical framework constructed by clustering themes, and refining relationships. Interpretation was theory driven.

**Results:**

Using template analysis, we identified four core collaborative strategies: connectedness, situation awareness, physical communication, and reflective practice, all embedded in a foundation of trust. From these findings, we developed the Expert Dyad Framework (EDF), which characterizes relational and cognitive behaviors, comprising of collaborative micro-practices, essential to high-functioning clinical partnerships.

**Discussion:**

The expert dyad framework contributes a practice-informed conceptual tool for educators, highlighting dyadic collaboration as a developmental target for those conducting health professions’ education. This study extends existing models of teamwork by focusing on the micro-interactions that underpin team performance.

## Introduction

High-quality clinical care depends not only on technical proficiency but also on relational and cognitive expertise [1,2]. While teamwork is widely recognized as essential to patient safety, much of the literature and educational focus has concentrated on large team structures and discrete communication skills [3]. For example, Salas et al.’s Big Five model posits that effective teamwork rests on five core dimensions which include team leadership, mutual performance monitoring, backup behavior, adaptability, and team orientation, supported by coordinating mechanisms such as shared mental models, closed-loop communication, and mutual trust [4]. Gittell’s Relational Coordination framework emphasizes that high performance teamwork is demonstrated when relational ties (shared goals, shared knowledge, mutual respect) underpin frequent, timely, problem-solving communication across interdependent roles [5]. This emphasis, though valuable, may overlook a critical collaborative unit: the healthcare dyad. Whether it involves a surgeon and anaesthetist, a midwife and obstetrician, or a nurse and physician, dyads serve as the smallest functional unit of teamwork and may determine team performance [6].

Emerging research in surgery and acute care highlights how dyadic familiarity (the dyad’s experience and frequency of working together) improves clinical outcomes, including reduced morbidity and greater workflow efficiency [6,7]. Yet while we are beginning to understand that dyads matter, far less is known about what makes them function at their best; and even less about how to support learners’ development of knowledge, skills, and attitudes related to dyadic collaboration. Most medical curricula continue to emphasize team-based or interprofessional simulations without explicitly addressing the nuanced behaviors and micro-practices that underpin high-performing dyadic partnerships [8].

This study explores the strategies established dyadic teams comprised of expert clinicians, use to coordinate care in high-acuity settings. Drawing on theories of relational coordination [9,10] and distributed cognition [11,12], we examine the cognitive and relational practices that allow dyads to function adaptively and in synchrony. Whereas in relational coordination theory, high team performance is generated from relationships grounded in shared goals, shared knowledge, and mutual respect, supported by frequent, timely, problem-solving communication, distributed cognition theory shifts the locus of cognition from the individual to the broader team-technology interface, proposing that thinking is distributed across people, tools, and environments [11,12,13].

**Figure 1:**
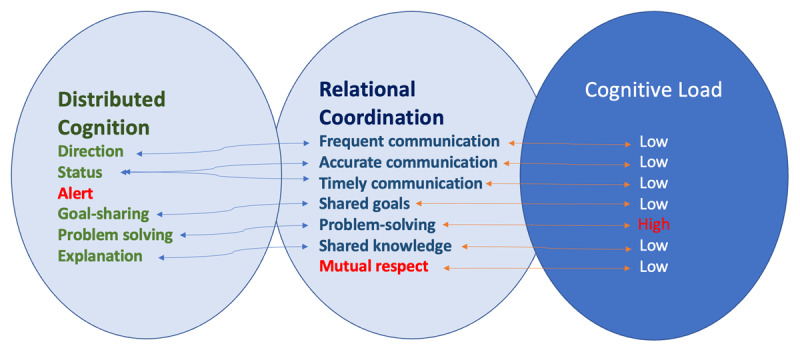
Distributed Cognition, Relational Coordination and Cognitive Load in Collaborative Teamwork.

Figure 1 presents our integrated conceptual model of dyadic collaboration, illustrating how a system of distributed cognition supports high-performing pairs. Within this framework, relational coordination is characterised by frequent, timely and accurate communication, shared goal setting, problem-solving and mutual respect. These behaviours enable the dyad to distribute cognitive work across partners and artefacts, thereby reducing individual cognitive burden (i.e., intrinsic and extraneous cognitive load) [14]. Importantly, the model maps this relational-cognitive system to a cognitive-load axis: when relational coordination is strong, partners experience lower cognitive load, freeing capacity for adaptive performance; conversely, when coordination is weak, cognitive load rises [15]. Thus, the inclusion of cognitive load emphasises not only what effective dyads do (the coordination behaviours) but how efficiently they manage mental processing demands during high-stakes interactions.

By making visible, often invisible micro-practices, we aim to contribute to medical education by asking the following questions.

1. What are the collaborative micro-practices actively cultivated by expert medical dyads?
2. Based on these micro-practices, how might we construct a conceptual model for understanding collaboration in dyads?

We argue that training clinicians to work effectively in pairs, not just in teams, may be a critical step toward improving care quality, clinician well-being, and system performance.

## Method

### Research Design

We conducted a limited realist-perspective study using Template Analysis [16], a method well suited for exploring the nuanced collaborative micro-practices of healthcare dyads [17,18]. We developed a preliminary coding “template” (informed by relational coordination and distributed cognition), applied the template to the data, and then iteratively modified it (adding, deleting, reorganising codes) as further data were coded. While template analysis allows for flexible, iterative application of both inductive and deductive coding, it differs from other analytical approaches such as directed content analysis which begins deductively with a set of theory-derived codes and seeks to validate or refine existing frameworks, only allowing new codes where data does not fit the template [19]. Using template analysis allowed us to generate inductive codes which we agreed gave richer insight to our data.

### Theoretical Rationale and Sensitizing Frameworks

We selected relational coordination and distributed cognition as the apriori concepts for this thematic analysis to guide our study of expert healthcare dyads [11,20]. Relational coordination theory conceptualizes coordination not purely as task alignment, but as relational work: relationships characterized by shared goals, shared knowledge, and mutual respect, which support frequent, timely, accurate, problem-solving communication (the core relational coordination theory dimensions). In high-stakes, interdependent clinical environments, relational coordination helps explain how relational dynamics underpin performance [21].

Distributed cognition shifts the focus from cognition as individual mental work toward cognition distributed across people, tools, environment, and time (i.e. shared mental models, artifacts, interactivity) [11]. Especially in procedural or acute care settings, where clinicians coordinate with instruments, displays, checklists, and environmental cues; distributed cognition helps us see how dyads extend cognition beyond individual minds.

Together, relational coordination and distributed cognition permitted us to scaffold our template analysis: relational coordination alerted us to relational codes (e.g. trust, communication patterns, shared understanding) while distributed cognition prompted codes about external supports, artifacts, cueing, distributed memory, and shared mental models. In our iterative coding process, we remained open to generating themes not anticipated by either theory, using relational coordination and distributed cognition more as lenses to organize and interpret the themes. Later, in discussing the expert dyad framework which we have developed, we map how each collaborative practice both aligns with and extends these theoretical lenses, demonstrating how micro-practices (e.g. gesture, calibration) embody and enrich the model. Relational coordination and distributed cognition emphasize the interdependent, contextual nature of teamwork; aligning well with our aim to examine how expert dyads enact collaborative expertise.

### Research Setting and Participants

We recruited participants from acute care environments; specifically operating rooms (OR), intensive care units (ICU, NICU), emergency rooms (ED); and delivery suites within tertiary hospitals in Australia, Switzerland, and the United States. We used a multi-national design to more rapidly access high-performing dyads across Australia, Switzerland, and the United States thus enriching the study’s credibility and transferability. These diverse settings bring variation in training traditions, organizational norms, communication culture, and healthcare systems. Our goal was to uncover micro-practices of dyadic collaboration that are resilient across different contexts, while also identifying context-sensitive variations. The multi-country design mitigates local bias and facilitates theory generalisability. Ultimately, this approach strengthens our confidence that the Expert Dyad Framework (EDF) captures relational and cognitive behaviors broadly relevant to global medical education and clinical practice.

KW and MM reached out to these medical dyads through professional associations, both verbally and via email, outlining the research objectives, procedures, and the informed consent process. The participants responded either verbally or through email. The settings we chose are characterized by high clinical complexity and high prevalence of medical error, making them ideal contexts to study expert collaboration [22]. Twenty participants, forming 10 interprofessional dyads, were purposively selected based on external perception (peer review or reputation). Inclusion criteria included daily practice together for at least one year of shared work experience in an acute, multi-team environment. The dyadic teams were considered experts and high performing by their supervisors and facility leaders. We defined a high-performing dyad as one that demonstrated all ten competencies outlined in the Team FIRST framework by Greilich et al; including structured communication, mutual trust, reflection and debriefing [3]. They were also considered experienced and expert teams in their specialties. Table 1 summarizes participant demographics.

**Table 1:** Participant Demographics.


DISCIPLINES	CLINICAL ENVIRONMENT	GENDER	CONTINENT	EXPERIENCE TOGETHER AS DYADS IN YEARS	NUMBER OF PROCEDURES TOGETHER

Nurse Anesthetist	Operating Room	Male	Europe	15.0	>100

Cardiac Anesthetist	Operating Room	Male			

Anesthesiologist	Operating Room	Female	North America	4.0	50–100

Ear, Nose and Throat Surgeon	Operating Room	Female			

Obstetrician	Emergency Room	Female	North America	1.0	10–50

Emergency Physician	Emergency Room	Female			

Neonatal Nurse	Neonatal ICU	Female	North America	3.0	10–50

Neonatologist	Neonatal ICU	Female			

Midwife	Obstetrics Department	Female	Australia	2.5	10–50

Obstetrician	Obstetrics Department	Female			

Emergency Nurse	Paediatric Emergency Room	Female	Australia	14.0	>100

Emergency Physician	Paediatric Emergency Room	Male			

Midwife	Labor and Delivery Room	Female	North America	6.5	>100

Obstetrician	Labor and Delivery Room	Female			

Surgeon- Ophthalmologist 1	Operating Room	Male	Australia	1.0	>100

Anesthetist 1	Operating Room	Male			

Surgeon- Ophthalmologist 2	Operating Room	Male	Australia	6.0	>100

Anesthetist 2	Operating Room	Male			

Bariatric Surgeon	Operating Room	Female	North America	3.0	>100

Surgical Assistant	Operating Room	Female			


### Data Collection

We created a semi-structured interview guide comprising questions based on educational theorist, Peggy Dettmer’s work on learning theory. She suggests four domains: interpersonal, emotional, cognitive, and physical/psychomotor [23]. To investigate how team members’ behaviours depend on one another and how recurring behaviour patterns emerge, we employed Kolbe’s adaptation of circular questions, a technique borrowed from family-therapy and simulation settings [24]. The guide (Appendix 1) as suggested by Morrison 2015, was not pilot tested, but refined iteratively as interviews progressed [25].

KW and MM conducted interviews between May and November 2022. Two interviews were co-conducted to establish alignment on approach; the remainder were administered by KW. Interviews were conducted with dyadic partners together, to foster naturalistic interaction and shared storytelling [26,27]. Each session was conducted on the Zoom platform, was audio-recorded and auto-transcribed and lasted between 45–60 minutes. Interview topics included how the dyad managed trust, communication, coordination under pressure, and reflective practice.

Although participants came from different countries, disciplines, and contexts (which shaped how they described their dyadic collaborations), they all expressed mutual respect and willingness to share freely. This diversity enriched our findings by revealing both shared practices and setting-specific priorities. We treat this variation as a strength, expanding the interpretive scope rather than suggesting a single, monolithic model of dyadic practice.

### Data Analysis

We approached analysis using template analysis as a hybrid deductive–inductive method. We began with a preliminary coding template informed by relational coordination and distributed cognition theories, containing initial apriori codes such as shared goals, shared knowledge, coordination cues, distributed cognition, and communication patterns. The first three interview transcripts were independently open coded by KW, MM, and MA to identify emergent behaviors or language beyond the initial template (e.g. emotional regulation, boundary norms, gestural communication). In iterative analytic meetings, we compared these initial codes, merged overlapping constructs, refined definitions, and developed a codebook with about 15 mid-level codes. Each code was accompanied by memos documenting the rationale for inclusion and boundaries.

Using that refined codebook, team members coded subsequent transcripts, meeting regularly to resolve discrepancies, discuss edge cases, and revise the codebook when we saw new patterns. We maintained an audit trail of code changes, recorded interpretive decisions, and used reflexive discussion to surface how our backgrounds shaped coding. Once coding was complete, we grouped codes into higher-order categories (for example, “coordination behaviors “and “relational dynamics,”) and iteratively tested whether these categories could cohere into a small set of collaborative practices across dyads. Through this process, we converged on four practices which surfaced repeatedly that included Connectedness, Situation Awareness, Physical Communication and Reflective Practice, and meaningfully captured dyadic dynamic patterns.

In the final interpretive phase, we mapped these practices back to relational coordination and distributed cognition theory, interpreting how each practice embodies or nuances theoretical constructs (e.g. gestural cueing as physical communication, connectedness as trust in relational coordination). Analysis continued until the data were judged to be sufficiently rich and that we had an adequate depth of understanding across participants [28,29]. To promote trustworthiness, we used triangulation (multiple coders), detailed audit memos, exemplar quotations in results, and periodic reflexivity checks.

### Reflexivity

Here we describe how the lenses our author team brought to our study may have influenced what we saw, valued, or emphasized in this study. By reflexivity, we mean cultivating explicit awareness of the ways our own perspectives and professional roles shaped how we framed the study and interpreted the data.

KW’s background is in sports doubles teams, clinical and administrative healthcare teams, and the development of programs designed to optimise team dynamics, and brings a lens shaped by both performance-based and systems-oriented perspectives to this study. MA is an educational scientist and instructional designer whose work focuses on translating theoretical frameworks into practical curriculum design within health professions education. Her extensive experience in faculty development and instructional innovation has likely heightened her sensitivity to the educational potential of dyadic interactions—particularly in how such relationships contribute to shaping meaningful learning experiences and fostering professional identity development. JWR is a lifelong athlete, simulation educator and researcher whose expertise in and affinity for deliberate practice, reflection, and mastery learning both informed—and may have biased—her take on study design and data interpretation. MK’s extensive experience in co-leading and researching simulation trainings, debriefing, and faculty development has likely shaped her view on the role of trust, relational coordination and candour in dyadic interactions. MM is an Emergency Physician and Simulation Educator. His work has a specific focus on resuscitation efforts of patients in critical care situations as well as training to those situations over the last 15 years. This work has likely piqued interest and built awareness around aspects of dyadic relationships and their impact on performance in critical care environments. As an obstetrician, PWT works in dyads of differing composition, for instance with anaesthesiology colleagues and with clinical midwifes. In his role as vice-dean, he has a dyadic relationship with the operational manager for education. In his experience, the value of dyadic work is recognized but not often explicitly discussed.

### Ethical Approval

This study received ethics approval through the Biomedical Research Alliance of New York (BRANY), an AAHRPP accredited organization, file number #21-12-498-373(HHC).

## Results

From the analysis of participant interviews we generated four collaborative practices that described the experience of expert dyads in healthcare. The practices were nurturing connectedness, demonstrating collaborative situation awareness, deploying physical and gestural communication, and using reflexivity regularly in practice. Underlying all four was trust, which functioned as a foundational condition for collaboration.

### Trust (Foundational Theme)

Trust fostered partners’ abilities to perform confidently, grow psychological safety, and underpinned all other practices. Table 2 includes subthemes, and illustrative participant quotes from expert dyads, demonstrating trust as a foundation for effective collaboration.

**Table 2:** Trust in Dyads.


SUBTHEME	ILLUSTRATIVE QUOTE	DYAD

Trust as foundation	***“***Not that I don’t trust other people, it’s just you don’t know them, and I think, knowing people and them knowing a little bit about you, you know who they are, and you’re comfortable. That’s what is really beneficial in terms of a great-working relationship.”	Midwife and Obstetrician 1

Psychological safety	“I know that [John] trusts my judgment. If I didn’t trust [John], I wouldn’t be able to go to him and feel psychologically safe in saying what my feelings are, because I’m just a nurse to some people.”	Emergency Nurse and Emergency Physician

Fragility vs abundance of trust	“Because if you can’t trust the other person, then you’re not part of a team. You have two people working and you’re doing your job and second guessing their job.”	Ophthalmologist and Anaesthetist.1.


### Connectedness

We define connectedness in healthcare dyads as comprising shared experience, transparency, humour, and remaining calm under pressure. Although no published work defines connectedness exactly this way, our formulation is informed by constructs in relational practice (which emphasize transparent relational atunement) and psychological safety (which supports emotional regulation and openness). We also draw on team literature on humour, where Lingard in 2013 defines humour as part of the drama of professional socialism [30]. Table 3 includes subthemes, and illustrative participant quotes from expert dyads demonstrating connectedness as a foundation for effective collaboration.

**Table 3:** Connectedness in Dyads.


SUBTHEME	ILLUSTRATIVE QUOTE	DYAD

Familiarity	“Knowing people and them knowing a little bit about you is really beneficial in terms of a great working relationship.”	Midwife and Obstetrician 1.

Transparency	“He is able to speak out loud all of his thoughts, and because he says what is happening, everyone is able to talk out loud.”	Anaesthetist and Anaesthetic Nurse

Levity/humour	“We’ll text each other stupid memes; it doesn’t break the flow, but it just sort of resets us.”	Obstetrician and Emergency Physician


### Situation Awareness

Wright, 2004 defines situation awareness “as a person’s perception of elements in the environment, comprehension of that information, and the ability to project future events based on this understanding”. Dyads cultivated situation awareness in both the clinical and emotional context through shared mental models and the ability to “read the room” Table 4. Table 4 includes subthemes, and illustrative participant quotes from expert dyads, demonstrating situation awareness as a foundation for effective collaboration.

**Table 4:** Situation Awareness in Dyads.


SUBTHEME	ILLUSTRATIVE QUOTE	DYAD

Shared context	“Understanding the surroundings and understanding each other makes us able to handle patient care issues together.”	Obstetrician and Emergency Physician

Emotional atunement	“He understands how others are feeling in the room, initiating reflection if a process has broken down.”	Ophthalmologist and Anaesthetist 1.

Shared mental models	“Shared communication is the most important bit to generate shared mental models of what performance should be like, and why.”	Ophthalmologist and Anaesthetist 2.


### Physical Communication

Eye contact and gestural communication, touch, and body language enabled silent coordination, reassurance, and emotion regulation. Table 5 includes subthemes, and illustrative participant quotes from expert dyads, demonstrating physical communication as a foundation for effective collaboration.

**Table 5:** Physical Communication in Dyads.


SUBTHEME	ILLUSTRATIVE QUOTE	DYAD

Rapid coordination	“We can just look at each other; and you know how serious it is without saying anything.”	ENT Surgeon and Anaesthetist 1

Emotional support	“I put my hand on his shoulder and said ‘breathe’; it grounded us both.”	Emergency Nurse and Emergency Physician


### Reflective Practice

Reflection was embedded before, during, and after clinical tasks, supporting adaptation, shared learning, and psychological safety. Table 6 includes subthemes, and illustrative participant quotes, from expert dyads showing reflective practice as a foundation for effective collaboration.

**Table 6:** Reflective Practice in Dyads.


SUBTHEME	ILLUSTRATIVE QUOTE	DYAD

Pre-briefing	“We had a conversation about what the plan would look like; a brief huddle about the day.”	Neonatal Nurse and Neonatologist

Collaborative problem-solving	“Whenever there’s a grey area, we talk about it collaboratively, knowing we might have to change the plan.”	Ophthalmologist and Anaesthetist 1.

Debriefing	“I like it a lot when I go to my colleagues and say, ‘what do you think of our work?’. Just with a coffee at the end of the day.”	Anaesthetic Nurse and Anaesthetist


Although the data were compelling from a qualitative perspective, we were interested from a quantitative perspective to understand how often certain behaviours and attitudes were stated and if experience correlated with certain statements or opinions. We uploaded the interviews to ChatGPT 5.0 and asked how often certain attitudes and behaviours were stated and if years of experience correlated with certain statements or opinions. We found that certain opinions were repeatedly stated and Table 7 is a record of how many dyads (of the 10) strongly or meaningfully invoked each theme. These words were either mentioned explicitly or implicitly through sub-themes and played a central role in the conversations.

**Table 7:** Repetition of Themes among Dyads.


THEME	DYADS (OUT OF 10) WITH STRONG/CENTRAL MENTION

Trust	10/10

Physical communication	10/10

Situation awareness including shared mental models	10/10

Connectedness including complimentary behaviours and role synergy	10/10

Reflective practice including debriefing	9/10

Boundaries including no public critique and respectful norms	8/10

Emotional regulation including being calm under pressure and co-regulation	8/10

Psychological safety and safe feedback	7/10


Exploring these themes, we found the concepts of trust, physical communication, situation awareness, connectedness and reflective practice were nearly universal. These elements were “core pillars” in our dyad sample. Learning and debrief practices were very common, though sometimes informal or partially done. Psychological safety was mentioned regularly, though not universally, as some dyads spoke more explicitly about “safety to speak up” more than others. Boundary norms and emotional regulation were also quite common but less universal than the core pillars.

To demonstrate any patterns between familiarity, (described by the number of procedures performed together and length of experience together), and collaborative behaviours and attitudes, we created Table 8.

**Table 8:** Analysis of Patterns Stemming from Dyadic Experience.


RELATIONSHIP/PATTERN	ANALYSIS	CAVEAT/POTENTIAL CONFOUND

When there was greater dyad familiarity, there was a higher shared volume of work and more explicit physical and gestural cues were deployed.	In dyads that had worked many years or many procedures together (e.g. >100), there was more mention of subtle gestural coordination (eye cues, small gestures) than in dyads with shorter tenure.	This could be a visibility bias. For example; when people know each other well, they notice these cues.

When there was more experience, (such as years in a role) there was more talk about emotional regulation and calmness.	Older, more senior participants more often spoke of staying calm, hiding emotion, or co-regulating under pressure.	Senior clinicians might also feel more able to articulate these emotions, so reporting bias might play a role.

Dyads with a strong learning culture (simulation, debriefs) tend to also emphasize psychological safety.	Where debriefs, feedback, and structured learning were strong, participants more often spoke about being safe to speak up, making mistakes, or emotional processing.	It may be that the same relational qualities (trust, openness) grow when there is more experience together, ensuring more psychological safety.

In dyads with more cross-discipline overlap (e.g. surgeon and anaesthetist) there was stronger talk of shared mental models, planning, and decision alignment.	In surgical/anaesthesia dyads, participants often mentioned stress pre-case, and so planned alignment, intra-case updates, and spoke of adapting together.	These dyads may inherently require more coordination, so the theme is situationally more visible.


We found that there was meaningful variation in how often themes were repeated across dyads. The “core pillars” of trust, physical communication, situation awareness, connectedness and reflective practice were universal in our sample and themes such as psychological safety and emotional regulation seemed to vary with familiarity, setting norms, and how intentionally the dyad invested in relational practices (debriefs, feedback, etc.).

We were also interested to understand, of the collaborative practices described, how the relational and cognitive underpinnings were expressed by the participants. Table 9 describes the practices and the relational and cognitive behaviours and attitudes with sample indicators.

**Table 9:** The Collaborative Practices Table; Relational vs Cognitive Behaviours and Attitudes.


COLLABORATIVE PRACTICES TABLE — RELATIONAL VS COGNITIVE			

PRACTICE/STRATEGY	RELATIONAL (INTERPERSONAL, AFFECTIVE)	COGNITIVE/STRATEGIC (THINKING, COORDINATION)	SAMPLE INDICATORS/NOTES

Trust	vulnerability, reliability, moral alignment	predictability, transparency, consistency	Many dyads said “I trust that she’ll do X”; trust developed over time.

Connectedness/Rapport	banter, personal stories, empathy, checking in		Dyads often “like each other,” share non-clinical chat.

Shared Mental Models/Pre-planning		aligning mental models, “what if” scenarios, defining endpoints	Pre-briefs, discussing a plan before the case starts.

Situation Awareness	reading partner cues, attention to environment	anticipating changes, scanning for cues, updating the mental model	Nonverbal cueing, mid-case updates, coordinate and adaptation.

Physical Communication	eye contact, posture shifts, touch, gestures	using gestural cues to coordinate timing, signalling	Gestures, shoulder taps, glance cues, spatial orientation

Reflexivity Reflection/Debriefing/Feedback	safe voice, open discussion, emotional processing	micro-analysis, “what went well/what not,” feedback loops	Many dyads do after-action reviews and informal post-case talk.

Boundary Norms/Conflict Avoidance	norms about not criticizing publicly, respectful tone	rules about where/when disagreement is aired	“We don’t disagree in front of the patient,” private resolution

Emotional Regulation/Co-regulation	calming presence, managing partner’s emotional state	maintaining focus under stress, regulating cognitive load	Dyads mention staying calm, mutual support when tense

Role Clarification/Complementarity	recognizing each other’s domain, respecting autonomy	distributing tasks, determining who leads in which domain	“She handles X, I handle Y,” seamless role handoffs

Learning Practices (Simulation, Checklists)	shared humility, joint improvement	structured tools, checklists, simulation, measurement	Use of simulation, checklists, experimenting, adopting new methods

Conflict Management/Tension Resolution	Airing disagreement respectfully and repairing relational strain	Negotiation, boundary enforcement, feedback timing	Some dyads mention “heated discussion” and always with resolution discussion


These findings informed the development of what we have called the expert dyad framework (EDF) which captures the nuanced behavioral patterns and collaborative micro-practices that characterize high-performing clinical partnerships.

**Figure 2:**
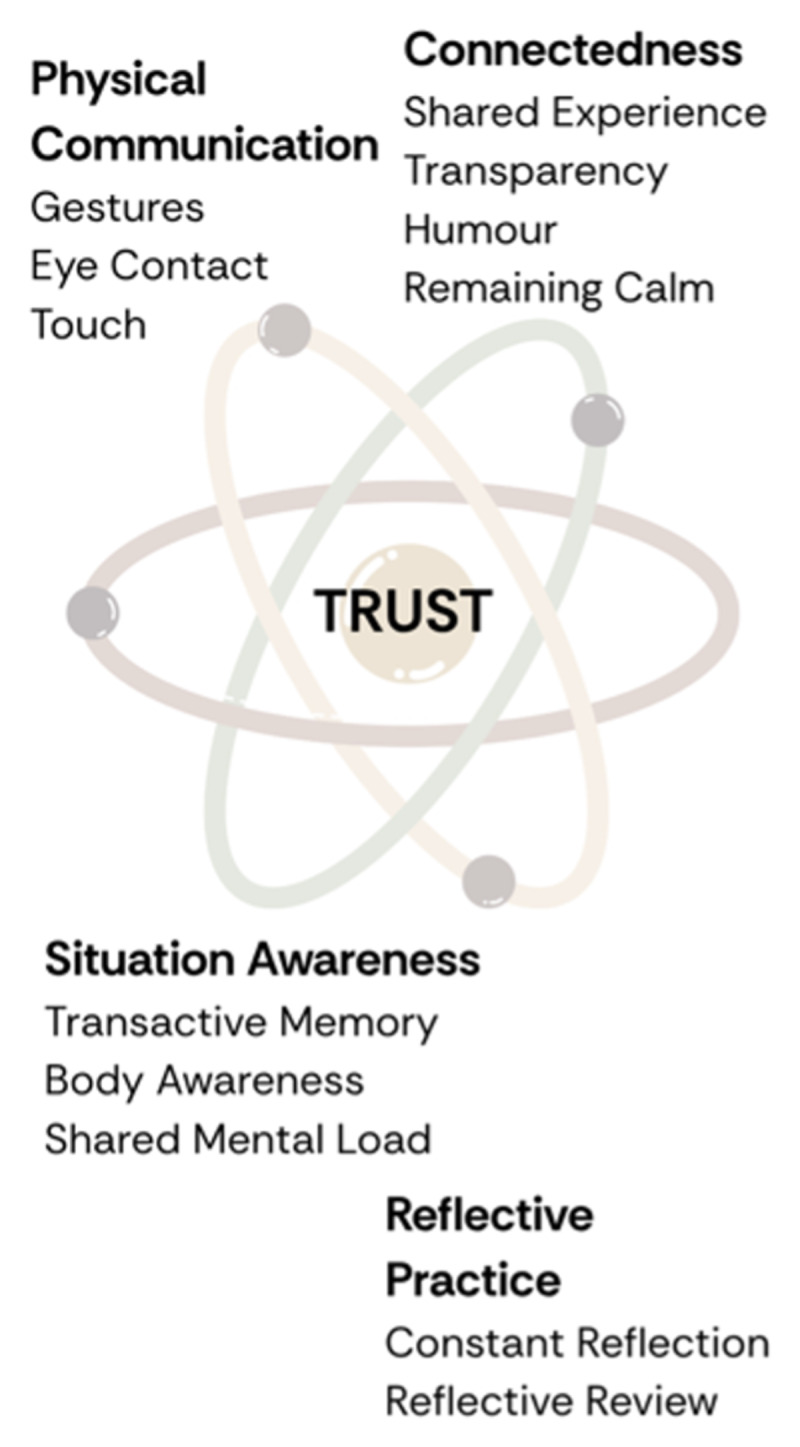
The Expert Dyad Framework.

Salas et al.’s Big Five model posits that effective teamwork rests on five core dimensions that include team leadership, mutual performance monitoring, backup behavior, adaptability, and team orientation, supported by coordinating mechanisms such as shared mental models, closed-loop communication, and mutual trust [4]. Gittell’s Relational Coordination framework emphasizes that high performance teamwork is demonstrated when relational ties (shared goals, shared knowledge, mutual respect) underpin frequent, timely, problem-solving communication across interdependent roles [5]. The EDF differs from both by zeroing in below the level of team and relationships to focus on moment-to-moment dyadic interactional micro practices (e.g. how two people negotiate turn-taking, repair, signalling). Where Salas captures team-level capacities and Gittell captures relational/communication infrastructures, EDF seeks to show how these are microprocesses between pairs in practice. See the EDF, Figure 2.

This framework zeroes in on the micro-practices deployed by expert health professions dyads working in acute settings and the behaviours and attitudes that embody these practices. During interviews the dyads described their experiences and Table 10 gives real examples of these micro-practices from the interviews.

**Table 10:** The Collaborative Micro-Practices used by Expert Dyads.


DYAD	COLLABORATIVE MICRO-PRACTICES	EXPERT DYAD FRAMEWORK CATEGORIES

Obstetrician and Emergency Physician	Including the other in early tasks	Situation Awareness

	Delivering reliably on commitments	Reflective Practice/Situation Awareness

	Frequent touchpoints/meetings	Physical Communication/Connectedness

	Naming expectations and reflecting together	Reflective Practice

Midwife and Obstetrician 1.	Sharing decisions together	Situation Awareness

	Gradually relinquishing control	Reflective Practice

	Reflecting on roles and affirming ownership	Reflective Practice/Connectedness

Emergency Nurse and Emergency Physician	Giving nurturing feedback	Connectedness/Reflective Practice

	Rotating through shared duties/exposure	Situation Awareness/Connectedness

	Social interaction (casual chats)	Connectedness

	Consistency in alignment of style	Physical Communication/Connectedness

Midwife and Obstetrician 2.	Consistent scheduling together	Physical Communication

	Observing shared mindset/approach	Situation Awareness

	Gradually letting the other take on tasks	Reflective Practice

	Adopting shared leadership norms	Reflective Practice/Connectedness

Ophthalmologist and Anaesthetist 1.	Debriefing item by item	Reflective Practice/Situation Awareness

	Discussing “why” behind standards	Reflective Practice

	Reflecting on system design & team flows	Reflective Practice/Situation Awareness

Ophthalmologist and Anaesthetist 2.	Asking “what happened?” in non-blaming way	Reflective Practice/Situation Awareness

	Talking through timing/micro adjustments	Situation Awareness

	Reflecting on team flow and patient experience	Reflective Practice/Connectedness

Surgical Assistant and Surgeon	Being paired consistently by management	Physical Communication/Connectedness

	Coordinating schedules	Physical Communication

	Senior scaffolding junior’s learning	Reflective Practice/Situation Awareness

	Junior gradually contributing fully	Reflective Practice

Anaesthetic Nurse and Anaesthetist	Intensive shared simulation training	Connectedness/Physical Communication

	Emotional/communication work in simulation	Connectedness/Reflective Practice

	Co-teaching over years	Situation Awareness/Connectedness

Surgeon and Anaesthetist	Collaborating early on a critical case	Situation Awareness/Connectedness

	Noting shared background/identity cues	Connectedness

	Explicitly resolving disagreements	Reflective Practice

	Maintaining openness through disagreements	Reflective Practice/Connectedness

Neonatal Nurse and Neonatologist	Informal hallway/incidental meetings	Physical Communication/Connectedness

	Observing caring gestures (notes, small touches)	Physical Communication/Connectedness

	Consistency of words and actions	Physical Communication/Reflective Practice

	Demonstrating care under pressure	Situation Awareness/Connectedness


The expert dyad framework offers a framework that extends current models of teamwork education by focusing on the micro-level dynamics that often go unaddressed in larger team training. While elements such as communication and reflective practice are not new to health professions education, our work emphasizes how these elements function specifically within dyadic interactions, where the stakes, intimacy, and interdependence are uniquely amplified [31]. Rather than duplicating existing models, the expert dyad framework draws attention to how trust, familiarity, and mutual support are enacted in real time. These insights gained from dyadic interviews can inform how we teach, assess, and support relational competence.

Conflict is a behaviour that requires attention when discussing high performing practices in medical dyads. In conversations with the participants, we found that conflict was less often named than other terms such as disagreement or maintaining boundaries. Many dyads preferred to frame ‘disagreement’ as discussion or negotiation, rather than ‘conflict.’ In many interviews, participants spoke of avoiding public conflict, they always engaged in private resolution, and often used micro-debriefs to manage small frictions rather than explicit ‘conflict management.’ The fact that conflict is rarely foregrounded may itself reflect cultural norms (e.g. in medicine, conflict is often handled subtly). Because we did not have ‘conflict management’ as a dedicated code originally, some nuances might have been subsumed under communications and boundaries.

## Discussion

In this study, we set out to explore the collaborative practices of expert healthcare dyads and consider how these relational and cognitive strategies could have an impact on teamwork training in medical education. By using the dyad as the unit of analysis, we identified four core practices; connectedness, situation awareness, physical communication, and reflective practice which were all underpinned by deep interpersonal trust.

For educators, the EDF can serve as a guide for designing medical education curricula that includes targeting dyadic collaborative skills, such as synchronized decision-making, gestural cueing, and mutual recalibration after unexpected events [32]. It can also support debriefing conversations that move beyond “what went wrong” toward “how did we read each other, adapt together, and reflect in action?” Additionally, the framework may inform the development of faculty tools for observing and coaching learners in dyad-based interactions; whether in clinical supervision, procedural tasks, or team-based care.

The growing emphasis on psychological safety and team resilience in medical education underscores the need to address the relational and cognitive foundations of teamwork [33]. Our findings suggest that strengthening dyadic function may be a powerful way to influence both individual and team performance. Just as trust has been shown to reduce critical errors and improve well-being, fostering dyadic trust through educational design may have cascading benefits for learners and patients alike [34] These behaviours align to Gormley & Nestel 2025, who describe how co-creating psychological safety through micro-communication skills in simulation-based education leads to psychological safety [35].

When we consider teamwork literature, there are a number of well-established teamwork frameworks, which emphasize competencies such as communication, mutual support, shared mental models, situation awareness, and debriefing (for example, TeamSTEPPS)[36]. The more recent Team FIRST framework identifies ten essential competencies; such as psychological safety, structured communication, closed-loop feedback, mutual performance monitoring, and reflection that are intended to underpin interprofessional team performance in healthcare [3]. These models are valuable and widely implemented, yet they tend to ignore the micro-practices between team members, specifying how two individuals coordinate in tightly coupled, high-stakes clinical interactions. Table 11 positions the Expert Dyad Framework within contemporary teamwork models, highlighting strengths and gaps for dyadic clinical coordination. In parallel, dyadic leadership models (e.g. in physician–administrator or clinical partnership contexts) emphasize role alignment, shared vision, and communication, but are oriented toward strategic or administrative functions rather than the moment-to-moment clinical domain [34]. The EDF aims to fill this gap by highlighting micro-level relational and cognitive practices; such as gestural cueing, emotional atunement, boundary norms about disagreement, and ongoing reflective recalibration that may be underarticulated in broader team or dyadic leadership models. The expert dyad framework offers a more granular, interactional lens for understanding how dyads achieve synchronous, safe, and adaptive collaboration in clinical contexts.

**Table 11:** Positioning the Expert Dyad Framework within contemporary teamwork literature.


FRAMEWORK	CORE FOCUS	STRENGTHS	LIMITATIONS/GAPS	EXPERT DYAD FRAMEWORK MICRO-PRACTICES

**Big Five Teamwork** (Salas, Sims & Burke, 2005)	Five core teamwork processes (team leadership; mutual performance monitoring; backup behaviour; adaptability; team orientation) supported by coordinating mechanisms (shared mental models; closed-loop communication; mutual trust)	High theoretical grounding; serves as a clear, parsimonious framework for team effectiveness across contexts; widely cited in the teamwork literature	Operates at team level rather than fine-grain dyadic micro-interaction; lacks direct focus on physical signalling, glance/gesture, micro-relational calibration in pairs	EDF complements this by specifying micro-practices like gestural cueing, relational calibration, emotional atunement, subtle boundary signalling between two interacting individuals

**TeamSTEPPS** King H, Battles J, Baker DP, Alonso A, Salas E, Webster J, Toomey L, Salisbury M. *TeamSTEPPS™: Team Strategies and Tools to Enhance Performance and Patient Safety*. Rockville, MD: Agency for Healthcare Research and Quality; 2006.	Standardized teamwork skills: communication, leadership, situation monitoring, mutual support, role clarity	Widely adopted; provides practical tools and training modules	Treats dyads as mini teams without specifying micro-coordination; limited focus on relational calibration or non-verbal signalling	Specifies gestural cueing, relational calibration, emotional atunement, boundary norms for disagreement and micro-adjustments mid-interaction.

**Team FIRST** Greilich PE, Kilcullen M, Paquette S, Lazzara EH, Scielzo S, Hernandez J, et al. Team FIRST framework: Identifying core teamwork competencies critical to interprofessional healthcare curricula. J Clin Transl Sci. 2023;7(1):e106.	10 teamwork competencies: recognizing teamwork, psychological safety, structured communication, closed loop, clarifying questions, sharing unique info, optimizing mental models, mutual trust, mutual performance monitoring, reflection/debriefing	Strong, evidence-based, tailored to healthcare; emphasis on interprofessional curricula	Operates at team level; less attention to how two individuals dynamically coordinate via gesture, glance, emotional regulation	EDF shows how Team FIRST competencies are embodied in dyads—e.g. how “structured communication” becomes gaze shifts or minimal cues; how “mental model alignment” becomes micro calibration and checking

**Frontiers Healthcare Team Effectiveness Framework** Zajac S, Woods A, Tannenbaum S, Salas E, Holladay CL. Overcoming Challenges to Teamwork in Healthcare: A Team Effectiveness Framework and Evidence-Based Guidance. *Front Commun*. 2021; 6:606445.	Broad challenges to teamwork in healthcare: accountability, conflict, decision making, reflection, communications, context (from qualitative + survey data)	Useful at system/team levels; identifies common barriers and enabling strategies	Doesn’ zoom into micro pair interaction; conflict and relational drift under-specified	EDF brings forward relational repair, micro-conflict norms, co-regulation under stress, and fine grain relational gestures

**Relational Coordination/Relational Perspectives** Gittell JH, Godfrey M, Thistlethwaite J. Interprofessional collaborative practice and relational coordination: Improving healthcare through relationships. Journal of Interprofessional Care. 2013;27(3):210–3.	Emphasis on work coordination via relationships: shared goals, shared knowledge, mutual respect, frequently timely communication	Excellent for linking relational and structural coordination at team/organizational levels	Less emphasis on *how* individuals enact relational coordination in minute-by-minute interaction	EDF shows how relational coordination is embodied: through glance cues, touch, micro reflection, mutual atunement in dyads

**Dyadic Leadership/Dyad partnership models** Clouser JM, Vundi NL, Cowley AM, Cook C, Williams MV, McIntosh M, Li J. Evaluating the clinical dyad leadership model: a narrative review. *J Health Organ Manag*. 2020;34(7):725–741	Role alignment, shared vision, trust, communication in leader–co-leader dyads (mostly administrative/strategic)	Focuses on pair relationships, role negotiation, trust	Usually distant from clinical micro-interaction, limited in capturing split-second cueing or emotional regulation in high pressure	EDF brings micro-relational responsiveness, calibration under pressure, feedback loops between two people, and micro experiential reflection in clinical dyads


Integrating the EDF alongside dominant team performance models (e.g. Salas’s Big Five with three coordinating mechanisms) reveals both convergence and innovation. The Big Five framework emphasizes leadership, backup behavior, adaptability, mutual performance monitoring, and team orientation, supported by shared mental models, trust, and closed-loop communication [4]. Our EDF aligns with many of these constructs; especially trust, shared mental models, and communication, and zeroes in on micro-relational dynamics (gestural calibration, dyadic connectedness and boundary norms) that are not fully specified in macro teamwork frameworks. Rather than merely extending an existing model, the EDF offers a fine-grain lens on how two individuals dynamically enact coordination under pressure.

## Strengths and Weaknesses of the Study

This study has several notable strengths. It addresses a critical gap in medical education literature by focusing on the healthcare dyad, a unit often overlooked in favour of larger teams, thereby offering novel insights into micro-level collaboration. The use of template analysis grounded in relational coordination and distributed cognition theory adds methodological rigor and theoretical depth. Additionally, the study’s international and interdisciplinary sample enhances the credibility and transferability of its findings. Furthermore, while rich in conceptual contribution, the EDF frames collaborative micro-practices that, although grounded in well-established teamwork constructs (e.g., shared mental models, relational coordination), are given a distinct emphasis through their application at the dyadic level. In other words, the EDF does not merely replicate existing team-performance frameworks but sharpens focus on the dyad as the unit of collaboration, providing a lens on relational and cognitive competence training that is under-explored in the literature. Nonetheless, its emphasis on dyadic dynamics offers an important pedagogical perspective that could enhance relational and cognitive competence training in clinical education. The EDF offers a practical model for educators seeking to enhance medical education programs through design simulation and feedback strategies that target dyadic performance.

However, the study also has limitations. The small sample size (10 dyads) may constrain the generalizability of its conclusions, and participants were identified through nomination, which could introduce selection bias.

While this study focused on experienced clinicians, we believe the insights are equally relevant for early learners. Introducing trainees to the principles of dyadic collaboration such as how to build trust, remain attuned to gestural signals, and reflect collaboratively, may enhance their capacity to learn and perform in interdependent roles. Future research could explore how to incorporate the expert dyad framework into curricula, how dyadic training adds to traditional team-based approaches, and how the framework may apply in other performance-critical domains such as aviation, sports, or emergency response.

## Conclusion

For medical educators, these findings suggest an addition to teamwork training by foregrounding dyadic collaborative skills, rather than focusing solely on larger team-level training. We recommend explicitly nurturing the relational and cognitive skills, that build interdependence and enable dyads to repeatedly function at a high level. Integrating dyadic behavioural skills into interprofessional education, including simulation and debriefing, offers a promising route to fostering relational competence, and adaptive coordination. As clinical systems grow more complex, equipping clinicians to partner effectively in pairs may be an essential, though frequently underemphasized, step toward building resilient, high-functioning teams.

## Additional File

The additional file for this article can be found as follows:

10.5334/pme.1932.s1 Appendix 1. Interview guide.
