# Supplementary material for: Collaborative Micro-Practices of Expert Healthcare Dyads: Implications for Medical Education
Source: Perspect Med Educ. 2026 Jan 20;15(1):1–15. doi: 10.5334/pme.1932 (PMC12829451; doi:10.5334/pme.1932)
Supplement: Appendix 1. — Interview guide. [file pme-15-1-1932-s1.pdf]

LEARNING PRACTICES SURVEY (for expert Tennis Doubles Players and Healthcare Dyads)

Introduction:

- Welcomed dyad participants to the interview and introduced myself and another interviewer if present.
- Explained the study goals – to explore the collaborative practices of expert dyads.
- Explained I would start with some demographic questions.
- Explained that the questions comprised four domains and circular questions;
    1. Social/interpersonal
    2. Affective/emotional
    3. Psychomotor/physical
    4. Cognitive
    5. Circular questions
- Explained the interview will be recorded and kept safe and secure. Only colleagues who are working on this project will have access.
- Study identifiers; explained we will not use your names or other identifying information and all your information will only be used for research purposes

Demographics:

Individual:

- What is your age? (20-35, 35-50, 50+)
- Experience in years?
- What is your position in your clinical unit? (not for tennis doubles)

Dyad:

- How long have you played/worked together?
- (Medicine)How many medical procedures have you performed together (e.g., <10, 10-50, 50-100, >100)

Social/interpersonal Domain

- You are an excellent dyadic team. What is it about the other person in the dyad that makes you perform better?
- Anecdotally, I have heard tennis players/clinicians say that trust is often regarded as an important part of a high functioning dyad. Tell me about your thoughts on trust and performance?
- What about your tennis/working relationship makes you effective in what you do?
- When you think about playing/working with your dyad partner, what things work more effectively than when you play/work with others?
- Think about a high stakes situation that happened when you were playing/working with your dyad partner. What qualities of the other person did you draw from to navigate that situation? How did you learn to draw upon those qualities?

Affective/emotional Domain

- I'm wondering if you could think back to that same high stakes' situation, what feelings or emotions around this do you recall?
    - How did you process your *feelings* towards the event and each other?

Psychomotor/physical Domain

- I notice that in high performing sports doubles teams there seems to be a lot of physical contact. What are your thoughts about physical contact in dyad partnerships?
- There are many non-verbal forms of collaboration. These include:
    - gesturing
    - pointing
    - making eye-contact; and
    - hugging.

  What are your thoughts on these non-verbal methods of collaboration? Did you have any deliberate conversations about your non-verbal conversations?

Cognitive Domain

- When making decisions, what helps you both decide together which is the best way forward?
- Can you tell me about your learning practices in these 4 areas?

    - A simulated or actual match/procedure with debriefing (includes prep/procedure/debriefing)
    - Deliberate practice of difficult and selective parts of a match/procedure.
    - Using and giving immediate feedback to each other and others.
    - Using any other resources such as checklists and videos.
- Looking back to when the two of you started playing/working together - how did **you get to** where you are now? How long did it take, how many times did you need to play/work together? How **did you learn to get** to where you are now?"
- How do you select learning practices to improve your performance both individually and in consultation with each other?
- "How do you decide when and how to improve performance?"

Circular Questions

- If other teams saw you two playing/working together, what would they see you do that makes you so effective as a team?
- If other teams saw you two playing/working together, what would they see that you don't do that makes you so effective as a team?

Thank you so much for your time. Can I call you again if I need further clarification.
Thank you.
